# A Hormone Receptor-Based Transactivator Bridges Different Binary Systems to Precisely Control Spatial-Temporal Gene Expression in *Drosophila*

**DOI:** 10.1371/journal.pone.0050855

**Published:** 2012-12-11

**Authors:** Shu-Yun Kuo, Chiao-Hui Tu, Ya-Ting Hsu, Horng-Dar Wang, Rong-Kun Wen, Chen-Ta Lin, Chia-Lin Wu, Yu-Ting Huang, Guan-Shieng Huang, Tsuo-Hung Lan, Tsai-Feng Fu

**Affiliations:** 1 Graduate Institute of Biomedicine and Biomedical Technology, National Chi-Nan University, Nantou, Taiwan; 2 Department of Applied Chemistry, National Chi-Nan University, Nantou, Taiwan; 3 Institute of Biotechnology and Department of Life Science, National Tsing Hua University, Hsinchu, Taiwan; 4 Department of Biochemistry and Graduate Institute of Biomedical Sciences, College of Medicine, Chang-Gung University, Taoyuan, Taiwan; 5 Department of Computer Science and Information Engineering, National Chi-Nan University, Nantou, Taiwan; 6 Department of Psychiatry, School of Medicine, National Yang-Ming University, Taipei, Taiwan; 7 Department of Psychiatry, Taichung Veterans General Hospital, Taichung, Taiwan; University of Houston, United States of America

## Abstract

The GAL4/*UAS* gene expression system is a precise means of targeted gene expression employed to study biological phenomena in *Drosophila*. A modified GAL4/*UAS* system can be conditionally regulated using a temporal and regional gene expression targeting (TARGET) system that responds to heat shock induction. However heat shock-related temperature shifts sometimes cause unexpected physiological responses that confound behavioral analyses. We describe here the construction of a drug-inducible version of this system that takes advantage of tissue-specific GAL4 driver lines to yield either RU486-activated LexA-progesterone receptor chimeras (LexPR) or β-estradiol-activated LexA-estrogen receptor chimeras (XVE). Upon induction, these chimeras bind to a LexA operator (*LexAop*) and activate transgene expression. Using GFP expression as a marker for induction in fly brain cells, both approaches are capable of tightly and precisely modulating transgene expression in a temporal and dosage-dependent manner. Additionally, tissue-specific GAL4 drivers resulted in target gene expression that was restricted to those specific tissues. Constitutive expression of the active PKA catalytic subunit using these systems altered the sleep pattern of flies, demonstrating that both systems can regulate transgene expression that precisely mimics regulation that was previously engineered using the GeneSwitch/*UAS* system. Unlike the limited number of GeneSwitch drivers, this approach allows for the usage of the multitudinous, tissue-specific GAL4 lines for studying temporal gene regulation and tissue-specific gene expression. Together, these new inducible systems provide additional, highly valuable tools available to study gene function in *Drosophila*.

## Introduction

The GAL4/*UAS* system has been widely used to regulate gene expression for functional studies in *Drosophila* and has been instrumental in making *Drosophila* one of the most genetically tractable model organisms. GAL4 gene trap or enhancer trap lines are generated via the random insertion of transposons into the *Drosophila* genome resulting in the disruption of gene expression [1], [2]. A large number of GAL4 driver lines exist and these have been of great value both in screening for insertional mutations as well as providing a powerful means to control targeted gene expression in specific *Drosophila* tissues [3].

The TARGET system combines a temperature-sensitive form of GAL80 (GAL80^ts^) and the GAL4/*UAS* system, allows for the control of gene expression at a specific time by a change in temperature [4]. The GAL80^ts^ temperature-sensitive mutant protein that blocks GAL4 activity at 18°C, the permissive temperature, while at 30°C, the restrictive temperature, GAL4 is not repressed since the GAL80 protein is inactive. However, temperature changes can result in unintended physiological effects including alterations in aging or lifespan [5], [6], [7], [8], [9], circadian rhythm [10], [11], [12], [13], [14], sleep [15], reproduction [16], [17], development [18], [19], [20], [21], learning and memory [19], [22], olfactory perception [23], and can induce mutations [24]. Furthermore, our own study found that some *UAS* lines have “leaky” gene expression at higher temperatures resulting in confounding GAL4 independent phenotypes.

Therefore, other strategies for controlling target gene expression at specific times in *Drosophila* have been developed, including hormone-mediated GAL4 activation approaches that include a chimeric GAL4, GAL4-ER, and GeneSwitch systems [25], [26]. In these systems the addition of exogenous molecules such as diethylstilbestrol (DES) or β-estradiol increases GAL4-ER activity [25], and mifepristone (RU486) induces the GAL4 activity of GeneSwitch [26]. Both systems transactivate target gene expression via the *UAS* when transgenic flies consume fly food containing hormones. Although the GeneSwitch system is more common now and GeneSwitch-enhancer trap lines have been developed in recent years [27], [28], it lacks the rich spatial variety that traditional GAL4 drivers provide.

In other organisms, hormone-induced trans-activation is mediated via LexA-fusion proteins. LexPR is a fusion product of a LexA-binding domain (LexA-BD), a p65 activation domain (AD), and the ligand-binding domain (LBD) of the human progesterone receptor [29]. Similarly, XVE is a product of the fusion of the LexA-BD, VP16 AD, and the LBD of the human estrogen receptor [30]. The regulatory mechanisms employed by these fusion products are similar to the GeneSwitch and GAL4-ER systems in that the inducers RU486 and β-estradiol tightly control the activity of LexPR and XVE, respectively.

**Figure 1:**
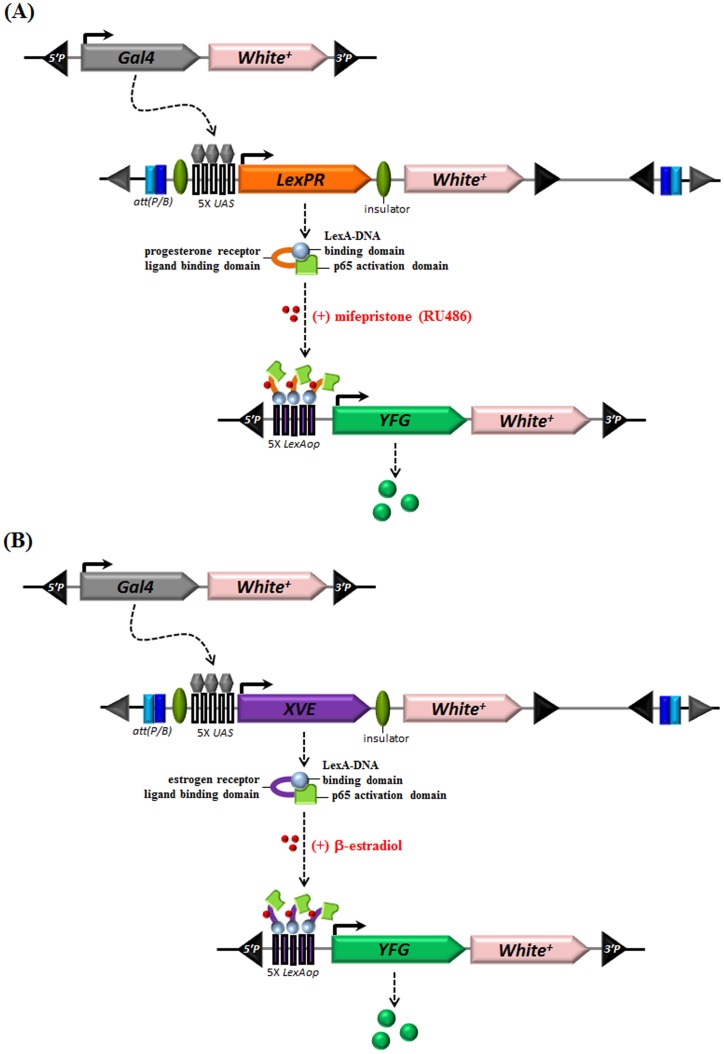
Analysis of *UAS-*Luciferase Lines to Identify Loci with Low Basal Luciferase Activity After Different Temperature Treatments. Luciferase activity was measured in 16 tests of 3 adults each. Activity was normalized to total protein levels and is shown as arbitrary units (a.u.). Each bar represents the mean (n = 16 for each group), and the error bars represent the standard error (± s.e.). Luciferase activity was measured in heterozygous *UAS*-luciferase transgenic flies without a Gal4 driver. (A) Gray bars indicate the luciferase activity of 6-day-old flies reared at 18°C. (B) Black bars indicate the luciferase activity of 2-day-old flies reared at 18°C that were exposed to 25°C for 2 days and then to 30°C for 2 days. White bars indicate the luciferase activity of 2-day-old flies reared at 18°C that were exposed to 30°C for 4 days.

Here we introduce both the LexRP/*LexAop* and XVE/*LexAop* conditional gene expression systems that are linked to the Gal4/*UAS* system in *Drosophila* as alternative approaches for the conditional control of gene expression (Figure 1). The new inducible systems incorporate the large array of existing Gal4 drivers to tissue-specific expression of the transactivators, LexRP/*LexAop* and XVE/*LexAop*, which can be conditionally regulated (turned on and off) by hormone inducers to further regulate transgene expression in flies. We demonstrate that both systems can precisely and tightly regulate a reporter gene expression specifically in a time- and dosage-dependent manner in fly brain cells. In addition using these systems we were able to reproduce the results of previous behavioral studies in which a constitutively active form of the mouse protein kinase A (PKA) catalytic subunit was used to alter sleeping patterns. Since the spatially and temporally precise control of gene expression is crucial to understanding complicated biological functions, our new inducible systems provide an additional experimental approach that will be useful for future *Drosophila* research.

## Results

### Identification of attP Sites that Support the Precise Regulation of Transgenes Following a Temperature Shift

The modified GAL4/*UAS* system (the TARGET system) can be conditionally regulated by a temperature-sensitive allele of *GAL80* (GAL80^ts^). At lower, permissive temperatures, GAL80 inhibits gene expression from the *UAS* promoter whereas, following a temperature shift, GAL80 is inactivated thereby allowing for the induction of GAL4-regulated gene expression. To determine whether shifting temperatures leads to leaky (Gal4-independent expression) transgene expression and to assess which existing attP sites are least susceptible to these effects, the induction of a series of lines containing different attP loci with *UAS*-luciferase reporter insertions were measured under the same temperature regime as used in the TARGET system.

**Figure 2:**
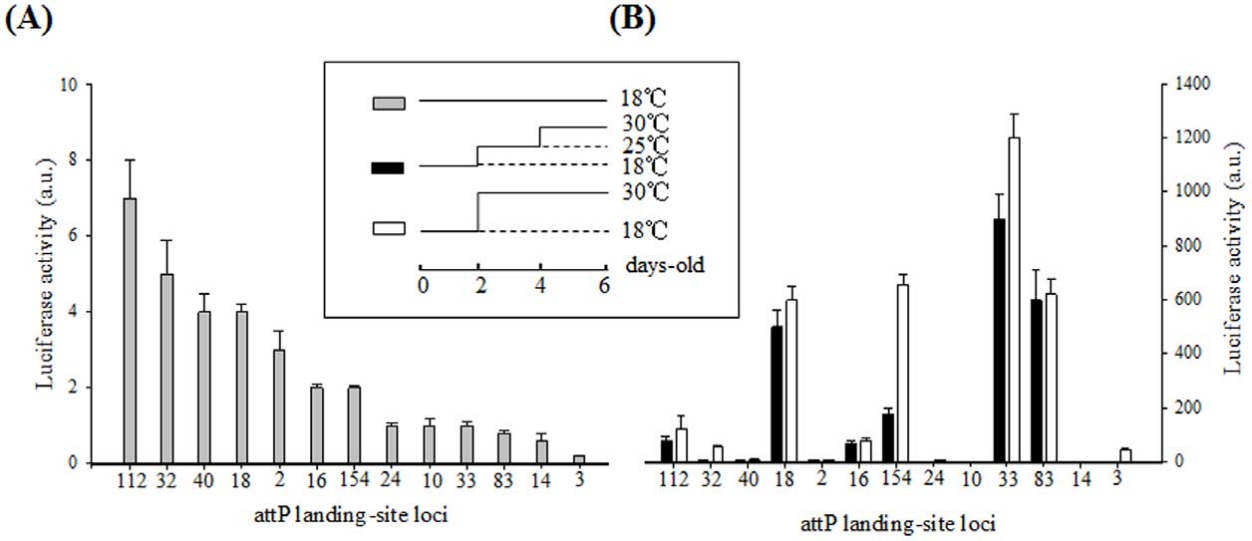
LexPR or XVE as Bridges for Spatial Targeting of Gene Expression. In a single animal, 3 separate *p*-element constructs were combined. Two were used for the GAL4/*UAS* binary gene expression system to specifically express either the LexPR or XVE chimeric proteins in a specific area and one carried the target gene of interest under the control of the *LexAop* operator sequences (*LexAop*). (A) A schematic diagram of LexPR under the control of the yeast upstream activating sequence (*UAS*) with the GAL4 driver. LexPR is a chimeric protein that includes the LexA-DNA binding domain (LexA-BD) fused to the human progesterone receptor ligand-binding domain and the p65 (NFκB) activation domain. Following treatment with RU486, LexPR is activated and binds to *LexAop* to drive the expression of Your Favorite Gene (*YFG*) downstream. In the absence of RU486, the target transgene remains silent. (B) A schematic diagram of XVE under the control of the yeast *UAS* with the GAL4 driver. XVE is a chimeric protein that includes the LexA-BD fused to the estrogen receptor ligand-binding domain and the p65 activation domain. Following treatment with β-estradiol, XVE is activated and binds to *LexAop* to drive the expression of *YFG* downstream. In the absence of β-estradiol, the target transgene remains silent.

Basal levels of luciferase activity were detected from the heterozygous *UAS*-luciferase transgenic lines in the absence of a GAL4 driver (Figure 2A). Upon temperature shift, a dramatic change in luciferase activity was observed in the *UAS*-luciferase lines with the attP18, attP33, attP83, and attP154 landing sites (Figure 2B), suggesting that these sites may result in leaky expression when used to generate *UAS* transgenic lines. Interestingly, five *UAS*-luciferase lines with tightly expressed loci (attP2, attP10, attP14, attP24, and attP40) maintained low levels of luciferase activity following temperature shift when compared to those that were not subjected to the shift, indicating that these constitute the optimal sites for transgene regulation using the TARGET system.

### Construction of the Hormone-Inducible LexPR and XVE Trans-activators to Tightly Regulate Transgene Expression in *Drosophila*

**Figure 3:**
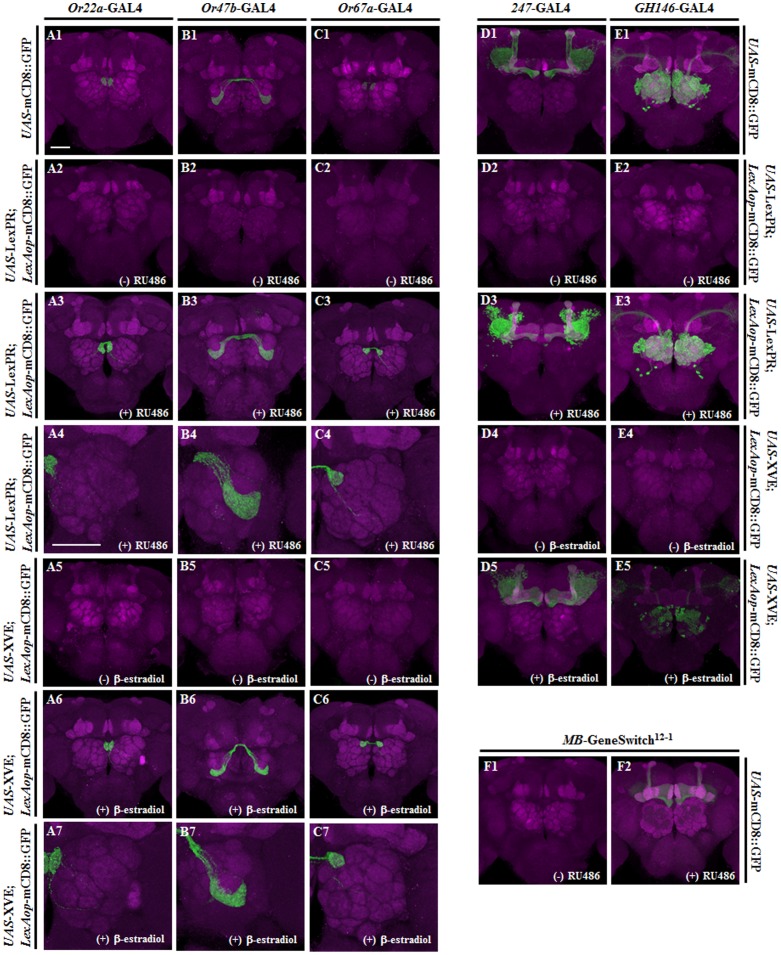
Induction and Activation of LexPR and XVE in the Adult Fly Central Nervous System. The trans-activation of LexPR and XVE was visualized in adult brains carrying the respective drivers and *LexAop*-mCD8::GFP. Confocal images are displayed of the *UAS*-mCD8::GFP expressed directly by several Gal4 drivers (A1–E1); *UAS*-LexPR acting as a bridge in the absence of RU486 (−) (A2–E2); *UAS*-LexPR acting as a bridge in the presence of 1.5 mM RU486 (+) for 5 days (A3–E3 are whole-brain images and A4–C4 are focused on 1 antennal lobe); *UAS/*XVE in the absence of β-estradiol (−) (D4–E4 and A5–C5); *UAS/*XVE in the presence of 30 mg/mL β-estradiol (+) for 5 days (A6–C6 and D5–E5 are whole-brain images and A7–C7 are focused on 1 antennal lobe). All induction methods activated *LexAop*-mCD8::GFP expression. Genotypes: (A1) *Or22a*-Gal4>*UAS*-mCD8::GFP-attP2, (A2–4) *Or22a*-Gal4>*UAS*-LexPR-attP40; *LexAop*-mCD8::GFP-attP2, (A5–7) *Or22a*-Gal4>*UAS*-XVE-attP40; *LexAop*- mCD8::GFP-attP2, (B1) *Or47b*-Gal4>*UAS*- mCD8::GFP, (B2–4) *Or47b*-Gal4>*UAS*-LexPR-attP40; *LexAop*- mCD8::GFP-attP2, (B4–7) *Or47b*-Gal4>*UAS*-LexPR-attP40; *LexAop*- mCD8::GFP-attP2, (C5–7) *Or47b*-Gal4>*UAS*-XVE-attP40; *LexAop*- mCD8::GFP-attP2, (C1) *Or67a*-Gal4>*UAS*- mCD8::GFP, (C2–4) *Or67a*-Gal4>*UAS*-LexPR-attP40; *LexAop*- mCD8::GFP-attP2, (C4–7) *Or67a*-Gal4>*UAS*- XVE-attP40; *LexAop*- mCD8::GFP-attP2, (D1) *247*-Gal4>*UAS*- mCD8::GFP-attP2, (D2–3) *247*-Gal4>*UAS*- LexPR-attP40; *LexAop*- mCD8::GFP-attP2, (D4–5) *247*-Gal4>*UAS*- XVE-attP40; *LexAop*- mCD8::GFP-attP2, (E1) *GH146*-Gal4>UAS- mCD8::GFP-attP2, (E2–3) *GH146*-Gal4>*UAS*-LexPR-attP40; *LexAop*- mCD8::GFP-attP2, (E4–5) *GH146*-Gal4>*UAS*-XVE-attP40; *LexAop*- mCD8::GFP-attP2, (F1–2) *MB*-GeneSwitch^12-1^>*UAS*- mCD8::GFP-attP2. Scale bar, 20 µm.

Although the attP loci described above provide precise expression upon temperature shifts, random integration is the most common strategy used for transgene generation. Here we report a new approach using LexPR or XVE in linking the GAL4/*UAS* and *LexAop* responders to precisely control transgene expression upon hormone induction (Figure 1). To evaluate the stability and effectiveness of the different trans-activators and to compare these systems, we used confocal imaging to examine GFP expression by different, tissue-specific Gal4 drivers. The membrane mCD8::GFP marker expression is specific to the glomeruli of the antennal lobes when controlled by the *Or22a*-Gal4, *Or47b*-Gal4, and *Or67a*-Gal4 drivers (Figure 3A1–C1). Similarly, mCD8::GFP expression is specific to mushroom bodies (MBs) when controlled by the *247*-Gal4 driver (Figure 3D1) and in projection neurons when controlled by the *GH146*-Gal4 driver (Figure 3E1). Similar expression patterns of the reporter were observed when the same Gal4 drivers were used in combination with *UAS*-LexPR (at the attP40 site) that was induced with 1.5 mM RU486 for 5 days to transactivate *LexAop*-mCD8::GFP (at the attP2 site) (Figure 3A3–E3, A4–C4). Likewise the reporter gene expression pattern was also recapitulated when Gal4 lines were used to activate *UAS*-XVE (at the attP40 site) in combination with treatment with 30 mg/mL β-estradiol for 5 days (Figure 3A6–C6, D5–E5). In contrast, no GFP expression was detected by confocal imaging of the brains of control flies that were not exposed to inducers (Figure 3A2–E2, A5–C5 and D4–E4) indicating that LexPR and XVE tightly regulate GFP expression in response to the inducer.

In particular, the LexPR inducible system showed more robust GFP expression in the MBs compared to that of the GeneSwitch system (Figure 3D3 and 3F2). GFP was quantified by collecting Z series over a height of 8 µm beginning with the first slice of calyx and extending to the sixth slice, which covered part of the ipsilateral calyx of each sample (Figure S1A–C). We found that the GFP intensity of *MB-*GeneSwitch^12-1^>*UAS*-mCD8::GFP in MBs was lower than that of the LexPR inducible system (*P* = 0.006) and *247*-Gal4>*UAS*-mCD8::GFP (*P* = 0.015) (Figure S1D). Together, these results demonstrate that the new hormone-inducible LexPR and XVE trans-activator systems efficiently and tightly regulate transgene expression in *Drosophila*.

### RU486-inducible LexPR Mediates Transgene Expression in a Time- and Dose-Dependent Manner in *Drosophila*

**Figure 4:**
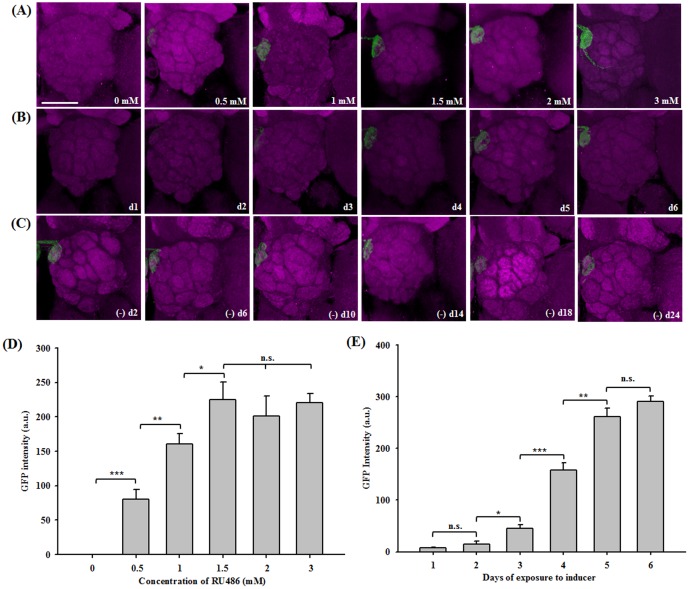
Time-course and Dose-response Analysis of the Inducible LexPR Bridge System in Response to RU486. (A) The trans-activation of LexPR was monitored in flies (*Or22a*-Gal4/*UAS*-LexPR-attP40; +/*LexAop*-mCD8::GFP-attP2) fed various concentrations of RU486 (0, 0.5, 1, 1.5, 2, and 3 mM) for 5 days. *LexAop*-mCD8::GFP expression was observed in one of the antennal lobes of adult brains. (B) *LexAop*-mCD8::GFP expression in flies fed 1.5 mM RU486 for 1–6 days (d1–d6). (C) The inducer was removed by replacing the food with fresh food for 2–24 days ((−) d2–d24). Using 3D projections, (D) the green fluorescence intensity of single glomeruli was analyzed in 5 samples from each group of induction by different concentrations of RU486 (0, 0.5, 1, 1.5, 2, and 3 mM) for 5 days, and (E) the green fluorescence intensity of single glomeruli was analyzed from 5 samples for each group of induction for 1–6 days. Each bar represents the mean, and the error bars represent the standard error (± s.e.). Data from each panel were analyzed using Student's *t* test, and any differences between various concentrations or treatment durations are indicated: n.s. indicates no significant difference; *** indicates p<0.001; ** indicates p<0.01; and * indicates p<0.05. Scale bar, 20 µm.

To evaluate the efficiency of the LexPR inducible system, we studied the kinetics of LexPR induction in *Or22a*-Gal4 lines with GFP expression in the DM2 glomeruli of the antennal lobes. Flies with the genotype *Or22a*-Gal4/*UAS*-LexPR; *LexAop*-mCD8::GFP/+ were fed media containing either 2% sucrose or 2% sucrose supplemented with RU486 at concentrations ranging from 0.5 to 3 mM for five days. The expression of *LexAop*-mCD8::GFP increased with increasing RU486 concentration and no GFP expression was detected in the absence of RU486 (Figure 4A). The GFP intensity of the DM2 glomeruli was used as a measure of trans-activation and that of individual DM2 glomeruli was analyzed using 3D projections. Following a five-day RU486 induction, the GFP intensity increased in a dose-dependent manner beginning with 0.5 mM RU486 and reaching a plateau at 1.5 mM RU486 (*P = *0.0004, no induction vs 0.5 mM; *P = *0.005, 0.5 mM vs 1 mM; *P* = 0.06, 1 mM vs 1.5 mM). No further increase in GFP intensity was detected at higher RU486 concentrations (*P* = 0.56, 1.5 mM vs 2 mM; *P* = 0.56, 2 mM vs 3 mM) (Figure 4D).

We also observed a time-dependent increase in the GFP signal following prolonged exposure of RU486 (Figure 4B), and this time-dependent increase was significant when comparing two days with increasing induction times (*P = *0.005, 2 days vs 3 days; *P = *0.0001, 3 days vs 4 days; *P* = 0.0013, 4 days vs 5 days). Six days of induction with 1.5 mM RU486 resulted in saturated GFP expression and no statistically significant increase relative to a five-day induction (*P = *0.17) (Figure 4E). These results indicate that LexPR tightly controls transgene expression in *Drosophila* in a time- and dose-dependent fashion in response to the RU486 inducer. These results also raised the question of whether GFP expression can be turned off upon inducer removal. After switching flies to food without RU486 on day seven, GFP expression is still strong by day twenty-four (Figure 4C) suggesting that the activated LexPR has stable and long-lasting activity or that membrane bound GFP is turned slowly after induction.

### LexPR and XVE Bridges Employ Different Binary Systems to Express the Constitutively Active PKA Subunit in MBs and Alter Sleep Patterns in *Drosophila*

A comparison of the three inducible systems (GeneSwitch, LexPR, and XVE) reveals that GFP expression following induction is observed in MBs at the same time (Figures 3F2, 3D3, and 3D5). To evaluate whether there are any functional differences in transgene expression among these inducible systems, sleep activity in different lines was measured. It has previously been shown that PKA expression in *Drosophila* MBs regulates sleep [31]. We used the constitutively active form of the mouse PKA catalytic subunit (mc*) [32] as the transgene to determine if each of the three systems can modulate sleep activity. We observed that PKA-dependent effects were facilitated by *UAS*-mc* that was induced using the GeneSwitch line (*MB*-GeneSwitch^12-1^/*UAS*-mc*-attP2), by *LexAop*-mc* that was induced using the *247*-Gal4 expressed LexPR (+/*UAS*-LexPR-attP40; *247*-Gal4/*LexAop*-mc*-attP2), and XVE (+/*UAS*-XVE-attP40; *247*-Gal4/*LexAop*-mc*-attP2) in MBs.

**Figure 5:**
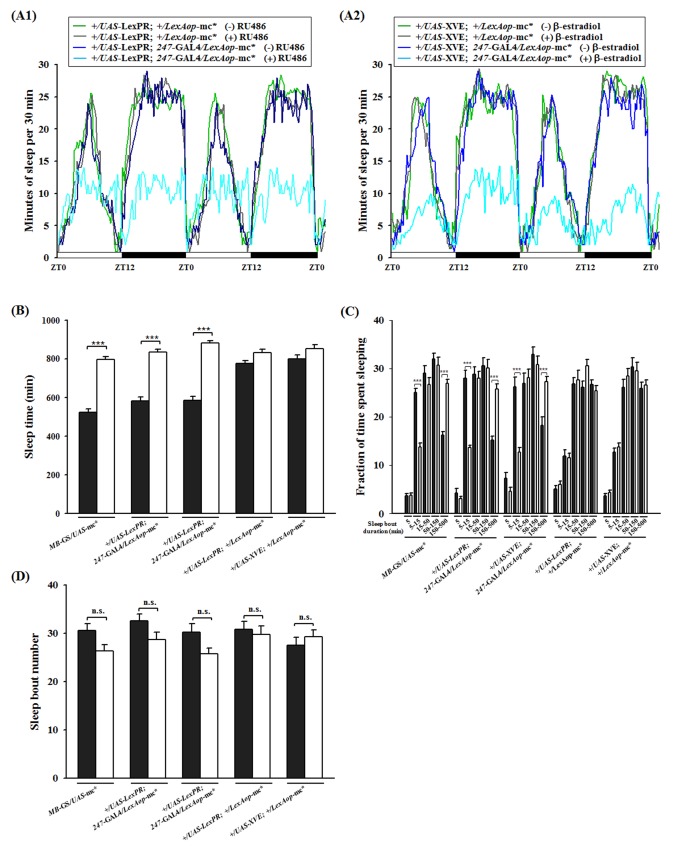
Expression of constitutively active PKA via activated LexPR (or XVE) modulates sleep in *Drosophila*. The effect of overexpression of the PKA catalytic activity in MBs on sleep behavior was measured using *UAS*-mc* that was directly controlled by the *MB*-GeneSwitch or indirectly controlled by LexPR/*LexAop*-mc* (or XVE/*LexAop*-mc*). (A1) Sleep was monitored in the absence or presence of 1.5 mM RU486. Genotypes: +/*LexAop*-LexPR; +/*UAS*-mc* (green line without inducer), +/*LexAop*-LexPR; +/*UAS*-mc* (gray line with inducer), +/*UAS*-LexPR; *247*-Gal4/*LexAop*-mCD8::GFP (blue line without inducer) and +/*UAS*-LexPR; *247*-Gal4/*LexAop*-mCD8::GFP (cyan line with inducer) (n = 32 for each group). (A2) Sleep was monitored in the absence or presence of 30 mg/ml β-estradiol. Genotypes: +/*LexAop*-XVE; +/*UAS*-mc* (green line without inducer), +/*LexAop*-XVE; +/*UAS*-mc* (gray line with inducer), +/*UAS*-XVE; *247*-Gal4/*LexAop*-mCD8::GFP (blue line without inducer) and +/*UAS*-XVE; *247*-Gal4/*LexAop*-mCD8::GFP (cyan line with inducer) (n = 32 for each group). After 6 days of induction with (black bars) or without (white bars) a suitable inducer, sleep behaviors were recorded for 2 days: (B) daily total sleep time, (C) sleep bouts in LD binned according to duration, and (D) the number of bouts. Data from each panel were analyzed using Student's *t* test, and any differences between various concentrations or treatment durations are indicated: n.s. indicates no significant difference; *** indicates p<0.001.

Sleep maintenance was altered by disruption of PKA homeostasis in MBs with a reduction in total sleep time and average sleep bout length (Figure 5A–C). Restricting the expression of the constitutively active form of the mouse PKA catalytic subunit in adult MBs significantly decreased daily sleep (Fig. 5A–B), which is consistent with an earlier report that examined a *MB*-GeneSwitch^12-1^>*UAS*-mc*-attP2 line with increased PKA activity [31]. Sleep time was significantly reduced in response to RU486 in *MB*-GeneSwitch^12-1^/PKA animals (*P*<0.0001) and *247*-Gal4/LexPR/PKA animals (*P*<0.0001), and in response to β-estradiol in *247*-Gal4/XVE/PKA animals (*P*<0.0001) (Fig. 5B). The percentage of short duration sleep episodes (5–15 min) increased (*P*<0.0001) and the long-lasting sleep episodes (150–500 min) decreased (*P*<0.0001) with disrupted PKA homeostasis in MBs (Figure 5C). These behaviors were unaffected in control flies that harbored the effectors even when the inducer was present (Figure 5A–C). However, there were no significant changes in the number of sleep bouts (Figure 5D). These data demonstrate that these systems work equally well in this assay, suggesting that this new approach for temporal regulation of transgene expression provides a better alternative and will be of great benefit to *Drosophila* research.

## Discussion

The TARGET system allows for gene expression at specific times and locations in *Drosophila* [4], [33]. However, a caveat of this method is that the necessary temperature shift may result in unintended effects on various biological functions. Alternative methods used to modulate transgene expression involve the addition of compounds or hormones rather than temperature shifts and include the tet-on and tet-off [34], [35], [36], the GAL4-ER, and the GeneSwitch systems [25], [26], [27], [37]. These methods, however, also have some drawbacks. For example in the fly model, the tet-on [38], tet-off [39], GAL4-ER [25], and GeneSwitch [26] techniques are mainly promoter-driven lines that do not display tissue specificity of transgene expression. Some researchers have successfully combined the tet-off (or tet-on) gene expression systems with the Gal4/*UAS* system for regulating gene expression [39], [40], [41]. However, the tet-off system uses a GAL4 driver to facilitate downstream *tTA* gene expression of *UAS* in specific tissues or cells and further controls target gene expression by the *tet* operator (*tetO*) upon exposure to doxycycline (Dox). While this modification overcomes the insufficient drivers in the tet-off system, it requires that flies continuously be fed Dox to maintain target gene expression. This treatment is not ideal since Dox is expensive and the long period of feeding may be harmful to the fly. The tet-off system is more effective than the tet-on system for regulating transgene expression [42] due, in part, to the lower transcriptional activity of rtTA compared with that of LexA::VP16AD and GAL4 [43]. More importantly, Dox has a stronger adverse effect on fly viability relative to RU486 after continuous exposure (Figure S2). Although the combination of Gal4/*UAS* and tet-off or tet-on allows for the precise expression of target genes, this approach is not optimal.

In this study GAL4 is used to induce either LexPR or XVE which, following induction with either RU486 or β-estradiol, respectively, results in the expression of a target gene downstream of *LexAop*. The results demonstrate that ingesting food that contained RU486 (or β-estradiol) enabled LexPR (or XVE) activation and trans-activation of the transgene downstream of *LexAop* in the transgenic flies. GFP was precisely expressed at the correct spatial location, and the fluorescence intensity was similar to the levels of LexPR (or XVE), indicating the precise specificity and high stability of the two new systems in regulating transgene expression in flies. As shown by measurements of the green fluorescence intensity, transgene expression levels can be regulated either by modulating the inducer concentration or the frequency of inducer feeding.

Hormone induction of transgene expression could be used to replace the induction by a change in temperature in the TARGET system to minimize the unexpected effects of temperature on behavior and development in *Drosophila*. Moreover, it can be used in combination with existing GAL4 drivers to provide ample options for tissue-specific expression. The combination of the new systems and the existing drivers overcomes the problem of insufficient tissue-specific drivers in the GeneSwitch system.

## Materials and Methods

### Flies

Flies were reared on standard, yeast-based fly food and housed in 12 h light–dark (LD) cycling incubators at 25°C and 50% humidity. The wild-type Canton-S strain was used. *247*-Gal4, *GH146*-Gal4, *Or22a*-Gal4, *Or47b*-Gal4, and *Or67a*-Gal4 lines were purchased from the Bloomington *Drosophila* Stock Center (BDSC) at Indiana University, and *MB*-GeneSwitch^12-1^ [44] was a gift from Dr. Ronald Davis (The Scripps Research Institute). *UAS*-luciferase flies (including insertions: attP2, attP3, attP10, attP14, attP16, attP18, attP24, attP32, attP40, attP83, attP112, attP154) flies were provided by Dr. Norbert Perrimon (Harvard Medical School, HHMI) [45]. *UAS*-LexPR-attP40, *UAS*-XVE-attP40, *UAS*-mCD8::GFP-attP2, *LexAop*-mCD8::GFP-attP2, *UAS*-mc*-attP2, and *LexAop*-mc*-attP2 flies were generated by injecting transgenes into embryos of 2U flies (*w^1118^* outcrossed 10 times with Canton-S) using a standard protocol for phiC31-mediated specific insertion on chromosome II attP40 landing loci or on chromosome III attP2 landing loci.

### Transgene Constructs

To reduce differential or leaky transgene expression resulting from positional effects, we re-generated *UAS* and *LexAop* fly lines by adding a gypsy insulator at both ends of the *UAS*-transgene and *LexAop*-transgene. We inserted the transgenes with phiC31 recombinase into attP2 or attP40 sites of flies [45]. First, the *UAS*-expression vector (p[PUAST-AI]) and *LexAop*-expression vector (pP[LexAop-AI]) with the attB site and gypsy insulators were constructed. Using pP[GreenPelican] as the starting vector, *Spe*I was used to cut pP[GreenPelican]. After filling in the cut site, the vector was further cut with *Sph*I to remove the eGFP gene. Similarly, *Bam*HI was used to cut the p[PUAST] vector, and after filling in the cut site, the vector was further cut with *Sph*I. The cut UAS_5X_ sequences included a mini-hsp70TATA promoter, multiple cloning sites, and an SV40 terminator. Ligation of the removed eGFP gene with pP[GreenPelican] was performed to form the pP[UAST-I] vector. Finally, attB from the pUASTattB vector was cut with *Spe*I and, after fill-in, was cloned into the filled *Tth111*I site of pP[UAST-I] to form the pP[UAST-AI] vector. The pP[UAST-AI-LexPR] construct was generated using standard molecular cloning procedures. Dr. Sergei Parinov (The National University of Singapore) kindly provided the pDs (Krt8:LPR-LOP:G4) clone containing single *Asc*I and *Sph*I sites for the release of the LexPR gene [29] and filling-in of the resulting blunt ends. This LexPR fragment was cloned into the filled-in *Not*I site of the pP[UAST-AI] vector to create the pP[UAST-AI-LexPR] transgene. The pP[UAST-AI-XVE] construct was generated by PCR amplification of the full coding region of XVE [30] with primers XVE-F (5′-ATGAAAGCGTTAACGGCCAGGCA-3′) and XVE-R (5′-TCAGACTGTGGCAGGGAAACCCT-3′). Dr. Nam-Hai Chua (Rockefeller University) kindly provided the pER8 clone as a template for PCR, and the amplified fragment was cloned into the pGEM-T Easy vector (Promega, Madison, WI). The pGEM-T Easy vector contains a single *Eco*RI site for the release of the XVE coding region. Following digestion of the XVE coding region, the fragment was then cloned into the *Eco*RI site of the pP[UAST-AI] vector to create the pP[UAST-AI-XVE] transgene. The pP[UAST-AI-mc*] and pP[LexAop-AI-mc*] constructs were also generated using standard molecular cloning procedures. Dr. Daniel Kalderon (Columbia University) kindly provided the pP[UAST-mc*] clone containing the *Kpn*I and *Xba*I sites for the release of the mc* coding gene, which encodes the constitutively active form of the mouse PKA catalytic subunit [32]. After cutting with *Kpn*I and *Xba*I, the mc* fragment was cloned into the pP[UAST-AI] and pP[LexAop-AI] vectors to create the pP[UAST-AI-mc*] and pP[LexAop-AI-mc*] transgenes, respectively.

### Inducing the Transactivation of LexPR and XVE

The transactivation of LexPR (or XVE) was observed via GFP expression of *LexAop*-mCD8::GFP in flies. The flies were starved for 12 h and then provided with food containing 5% sucrose, 2% agar, and either 1.5 mM RU486 or 30 mM β-estradiol for 6 days. Additionally, we also used GFP expression resulting from different doses of RU486 to confirm the trans-activation of LexPR. The 3-day-old flies (*Or22a*-Gal4 driver X *UAS*-LexRP-attP40; *LexAop*-mCD8::GFP-attP2) were reared with food containing RU486 (0, 0.5, 1, 1.5, or 2 mM) for 1–6 days. Fresh food with the same concentration of inducer was provided every 2 days. GFP expression was observed using a confocal microscope after induction.

### Heat-Shock Regimen and Luciferase Activity Assay

Several *UAS*-luciferase lines with confirmed low basal activity [45] were reared at 18°C. They were later exposed to one of three different temperatures so that any apparent leaky expression of luciferase activity due to temperature changes could be detected. After eclosing, the 6-day-old flies were collected and divided into 3 groups. One group was maintained at a fixed temperature of 18°C for 6 days. The second group was maintained at 18°C for 2 days and later at 30°C for 4 days. The third group was maintained at 18°C for 2 days, 25°C for 2 days, and 30°C for 2 more days. Three flies were collected from each treated group, and we used a steady-Glo® Luciferase Assay kit (Promega, Madison, WI) to measure luciferase activity. Flies were collected in 1.5-mL microcentrifuge tubes. Glo Lysis buffer (100 µL) (Promega, Madison, WI) was added to each tube, and samples were homogenized using a minibeadbeater-16 (Biospect products, Bartlesville, OK). Samples were then centrifuged at 4°C and 13000 rpm for 15 min. After centrifugation, 20 µL of the supernatant was transferred to a 96-well white wall plate. After adding 20 µL of Luciferase Luciferin Reagent (Promega, Madison, WI), plates were incubated at room temperature in the dark for 10 min. Fluorescence was measured by a luminometer (Perkin-Elmer TopCount NXT) (Perkin-Elmer Waltham, MA). A portion of each sample was taken for protein concentration analysis using a BCA Protein Assay Kit (Pierce, Rockford, IL) together with a standard for normalization. The protein assay was performed by diluting 25 µL of fly extract lysate 5 times with lysis buffer and transferring it to a 96-well plate. After the addition of BCA working reagent (200 µl), the plates were incubated at 37°C for 30 min. A spectrophotometer was used to measure the absorption at 562 nm.

### Bio-imaging and sample preparation

Fly brains were dissected in isotonic phosphate-buffered saline (PBS). Samples were fixed by incubating them in 4% *p*-formaldehyde and microwaving them in an ice bath for 3 min. Samples were then transferred to 0.25% Triton X-100 in 4% *p*-formaldehyde and microwaved for an additional 3 min in an ice bath. Fixed samples were immersed in penetration/blocking (P/B) buffer (2% Triton X-100, 0.02% NaN_3_, and 10% normal goat serum in PBS) and incubated in a vacuum for 10 min. The buffer was replaced with fresh P/B buffer, and the procedure was repeated 3 more times. Samples were then incubated in P/B buffer at room temperature for 2 h. After a brief rinse with PBS, the brains were counterstained with anti-DLG antibody at room temperature overnight. Samples were washed 3 times with wash buffer (2% Triton X-100 and 3% NaCl in PBS) for 20 min. After washing, samples were incubated with secondary antibody (anti-mouse-IgG conjugate biotin) (Invitrogen) overnight. After incubation, samples were washed 3 times with wash buffer for 10 min. Finally, samples were incubated in a solution containing Alexa Fluor 633 streptavidin (Invitrogen) overnight, washed 3 times with wash buffer for 10 min, mounted in Focusclear™ (CelExplorer Labs®, Taiwan), and examined using a Zeiss LSM 700 confocal microscope equipped with a 40× C-Apochromat water immersion objective lens.

### Sleep assay

To examine sleep activity, flies were collected 1 day after eclosion and placed in glass bottles in groups of approximately 100 with food containing 1.5 mM RU486 (or 30 mg/ml β-estradiol) for 5 days. After the induction period, flies were acclimated for approximately 24 h at 25°C in 12-h light/12-h dark (LD) conditions. Flies were then transferred to sleep assay tubes containing a suitable inducer and entrained for 1 day. Locomotor activity was recorded for 3 days in LD by using a *Drosophila* activity monitor (DAM) (TriKinetics, Waltham, MA) [46]. A sleep episode was defined as a 5-min bin of uninterrupted quiescence, as measured by the DAM system. Activity counts were summed across all wake bins and analyzed. We calculated total sleep time, average length of sleep episodes, and the number of sleep episodes.

## Supporting Information

Figure S1 **Quantification of GFP under the control of constitutive or inducible expression systems in the MB calyx.** The image gallery shows confocal Z-series images from posterior to anterior that include 8 µm stacks (merged) with 7 slices in the ipsilateral calyx of (A) RU486-activated *MB*-GS>*UAS*-mCD8::GFP; (B) RU486-activated *247*-Gal4>*UAS*-LexPR; *LexAop*-mCD8::GFP and (C) *247*-Gal4>*UAS* -mCD8::GFP. (D) Using 3D projections, the green florescence intensity of Z-stack confocal images was analyzed in 3 samples from each group. Each bar represents the mean, and the error bars represent the standard error (± s.e.). Data from each panel were analyzed using Student's *t* test, and any differences between various genetic manipulations are indicated: ** indicates p<0.01 and * indicates p<0.05. Scale bar, 20 µm.
(TIF)

Figure S2 **Increasing Dox but not RU486 concentration strongly correlates with fly lethality.** The indicated CS flies were maintained in the absence and presence of various concentrations of inducing drugs, and groups of flies were raised on appropriate dosages of Dox (0.25, 1, and 2 mg/mL) or RU486 (1 and 3 mM) for the indicated time course for 0–8 days. The percentage of surviving flies was calculated and plotted. The data points represent the means (3 tests of 210 adults each), and the error bars represent the standard error values (± s.e.). Lethality strongly correlated with increasing concentrations of Dox but not with increasing concentrations of RU486.
(TIF)
