# Correction: A Hormone Receptor-Based Transactivator Bridges Different Binary Systems to Precisely Control Spatial-Temporal Gene Expression in *Drosophila*

**DOI:** 10.1371/annotation/acecbee6-7464-463e-9a10-700f51e9c4f5

**Published:** 2013-07-15

**Authors:** Shu-Yun Kuo, Chiao-Hui Tu, Ya-Ting Hsu, Horng-Dar Wang, Rong-Kun Wen, Chen-Ta Lin, Chia-Lin Wu, Yu-Ting Huang, Guan-Shieng Huang, Tsuo-Hung Lan, Tsai-Feng Fu

Figures 1 and 2 were incorrectly switched. The image currently appearing as Figure 1 belongs with the title and legend for Figure 2, and the image currently appearing as Figure 2 belongs with the title and legend for Figure 1. 

