# Supplementary figures and images for: A Hormone Receptor-Based Transactivator Bridges Different Binary Systems to Precisely Control Spatial-Temporal Gene Expression in Drosophila
Source: PLoS One. 2012 Dec 11;7(12):e50855. doi: 10.1371/journal.pone.0050855 (PMC3519826; doi:10.1371/journal.pone.0050855)

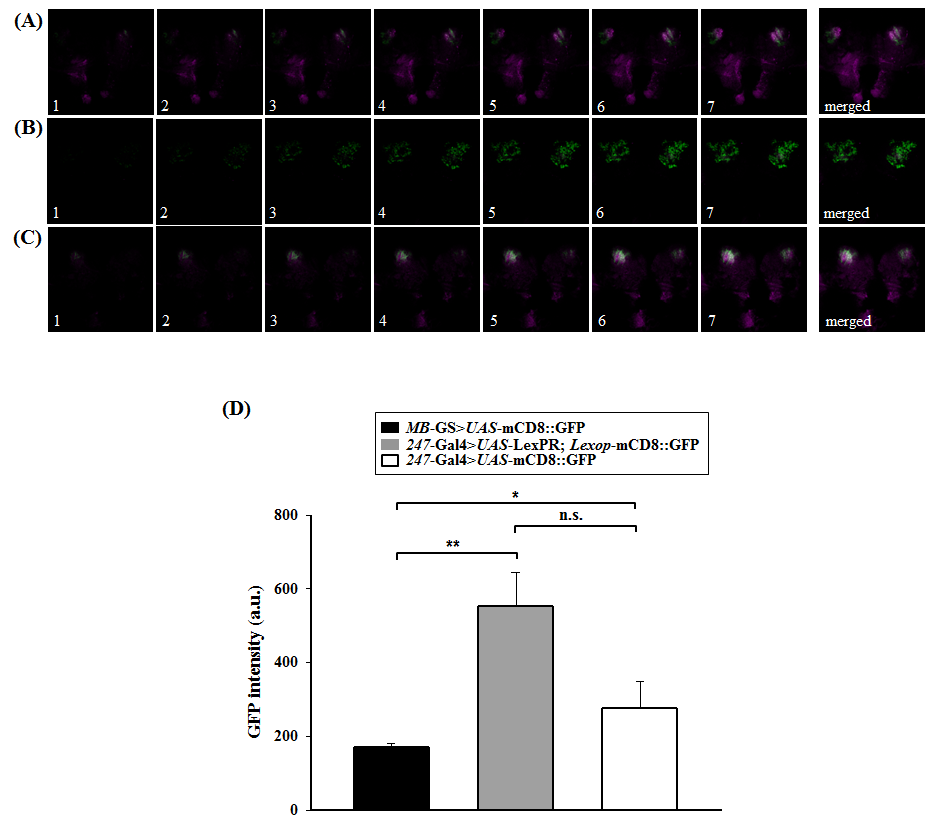

Supplement: Figure S1 — Quantification of GFP under the control of constitutive or inducible expression systems in the MB calyx. The image gallery shows confocal Z-series images from posterior to anterior that include 8 µm stacks (merged) with 7 slices in the ipsilateral calyx of (A) RU486-activated MB-GS>UAS-mCD8::GFP; (B) RU486-activated 247-Gal4>UAS-LexPR; LexAop-mCD8::GFP and (C) 247-Gal4>UAS -mCD8::GFP. (D) Using 3D projections, the green florescence intensity of Z-stack confocal images was analyzed in 3 samples from each group. Each bar represents the mean, and the error bars represent the standard error (± s.e.). Data from each panel were analyzed using Student's t test, and any differences between various genetic manipulations are indicated: ** indicates p<0.01 and * indicates p<0.05. Scale bar, 20 µm. (TIF) [file pone.0050855.s001.tif]

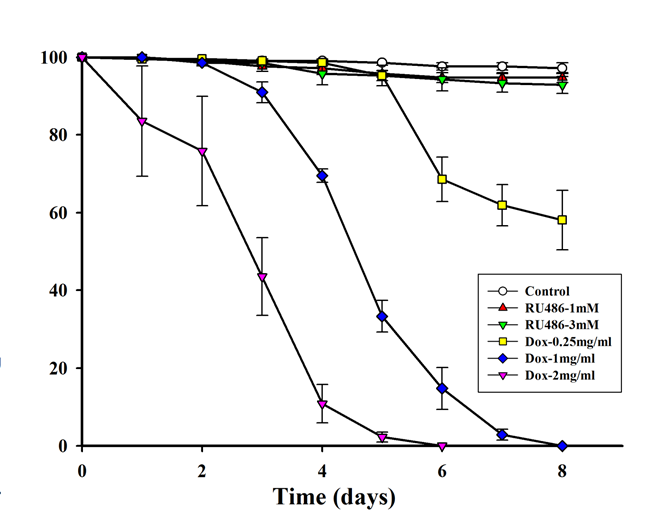

Supplement: Figure S2 — Increasing Dox but not RU486 concentration strongly correlates with fly lethality. The indicated CS flies were maintained in the absence and presence of various concentrations of inducing drugs, and groups of flies were raised on appropriate dosages of Dox (0.25, 1, and 2 mg/mL) or RU486 (1 and 3 mM) for the indicated time course for 0–8 days. The percentage of surviving flies was calculated and plotted. The data points represent the means (3 tests of 210 adults each), and the error bars represent the standard error values (± s.e.). Lethality strongly correlated with increasing concentrations of Dox but not with increasing concentrations of RU486. (TIF) [file pone.0050855.s002.tif]
